# Are meteotsunamis an underrated hazard?

**DOI:** 10.1098/rsta.2014.0377

**Published:** 2015-10-28

**Authors:** Charitha B. Pattiaratchi, E. M. S. Wijeratne

**Affiliations:** 1 School of Civil, Environmental and Mining Engineering, The UWA Oceans Institute, The University of Western Australia, Crawley 6009, Australia; 2 Bushfire and Natural Hazards Cooperative Research Centre, Melbourne, Australia

**Keywords:** meteotsunamis, Proudman resonance, Greenspan resonance, global

## Abstract

Meteotsunamis are generated by meteorological events, particularly moving pressure disturbances due to squalls, thunderstorms, frontal passages and atmospheric gravity waves. Relatively small initial sea-level perturbations, of the order of a few centimetres, can increase significantly through multi-resonant phenomena to create destructive events through the superposition of different factors. The global occurrence of meteotsunamis and the different resonance phenomena leading to amplification of meteotsunamis are reviewed. Results from idealized numerical modelling and field measurements from southwest Australia are presented to highlight the relative importance of the different processes. It is shown that the main influence that leads to amplification of the initial disturbance is due to wave shoaling and topographic resonance. Although meteotsunamis are not catastrophic to the extent of major seismically induced basin-scale events, the temporal and spatial occurrence of meteotsunamis are higher than those of seismic tsunamis as the atmospheric disturbances responsible for the generation of meteotsunamis are more common. High-energy events occur only for very specific combinations of resonant effects. The rareness of such combinations is perhaps the main reason why destructive meteotsunamis are exceptional and observed only at a limited number of sites globally.

## 1. Introduction

The major hazard in coastal regions is inundation by extreme water levels generated in the ocean by different mechanisms such as storm surges and tsunamis or through a combination of effects such as a relatively small storm surge coinciding with high astronomical tides. The impacts of seismic tsunamis (generated through underwater earthquakes) have been highlighted by the recent mega-tsunamis in the Indian Ocean (2004) and Pacific Ocean (2011). These events were accompanied by large loss of life and extreme damage to coastal infrastructure. Similarly, the effects of storm surges have had significant effects such as those due to major storms: *Katrina* in New Orleans; *Sandy* in New York City; and *Haiyan* in the Philippines. These events also highlighted the effects of coastal inundation with major impact on coastal infrastructure, albeit with a significantly smaller number of casualties mainly due to lead times associated with storm propagation.

**Figure 1.**
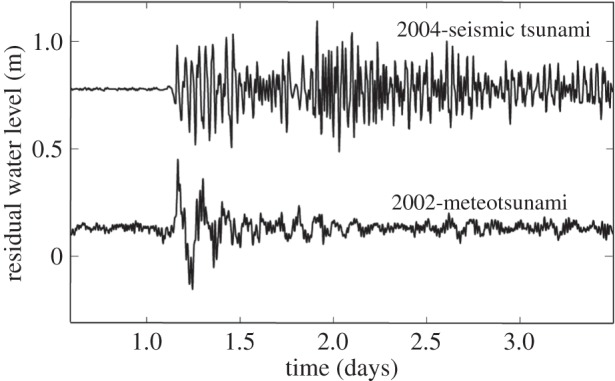
Time series of residual water level (filtered to include periods less than 6 h) recorded at Fremantle Boat Harbour during the 2004 Indian Ocean tsunami and a meteotsunami recorded in 2002.

**Figure 2.**
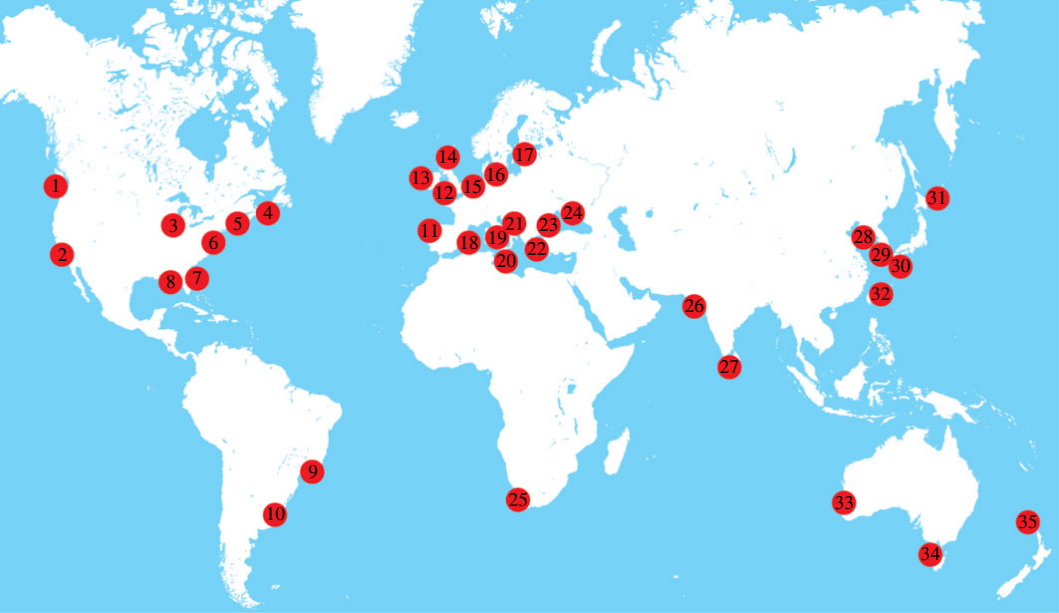
Locations of meteotsunami occurrences as reported in the literature. Numbers refer to references in table 2.

Meteorological tsunamis (meteotsunamis) are water-level oscillations which are similar to waves generated by seismic activity (‘tsunami waves’), except they have a meteorological origin and are not generated through seismic activity, volcanic explosions or submarine landslides [1,2,3,4]. Time series of water-level records from Fremantle (Western Australia) obtained during the seismic tsunami of 2004 and a meteotsunami in 2002 indicate similar wave heights for both events (figure 1). The first description of meteorological effects generating tsunamis was by Nomitsu [5], but the scientific interest in meteotsunamis has only increased over the past decade or so with reports of more widespread occurrences (figure 2 and table 2). The term ‘meteorological tsunamis’ was introduced by Defant [6] to define water-level fluctuations resulting from atmospheric phenomena such as squalls, thunderstorms, frontal passages and atmospheric gravity waves; however, the first reported work on meteorological effects generating tsunamis was published in 1935 [3,5]. The main forcing mechanism of a meteotsunami is the propagation of an abrupt change in sea surface atmospheric pressure and/or associated wind gusts. Recent work [1,3,7,8,9] and the occurrence of several events globally in the past few years have highlighted the importance of meteotsunamis as a coastal hazard similar to that of seismic tsunamis [10]. Although meteotsunamis are not catastrophic to the extent of major seismically induced basin-scale events, they have, nevertheless, caused millions of dollars of damage to boats and harbours around the world, and have claimed lives (seven people killed while fishing on a sunny day in Chicago, IL, in 1954; [11]). The main differences between seismic/landslide tsunamis, meteotsunamis and storm surges are shown in table 1.

**Table 1.** Contrasting characteristics of seismic tsunamis, meteotsunamis and storm surges (modified from [12]).

	seismic tsunamis	meteotsunamis	storm surges
generation mechanism and source location	from below the sea surface by sudden displacements of the sea floor	from above the sea surface by the inverse barometer effect coupled with resonance effects	from above the sea surface by the inverse barometer effect and a strong wind field
source duration	impulsive motions of the sea floor which last O(minutes)	driven by resonance effects which take effect O(hours)	driven by atmospheric pressure and wind field O(hours–days)
source extent	fixed to the region of uplift	propagate over the region	propagate over the region
water depth	occur in deep or shallow water, but higher waves are generated in depths greater than the continental shelf	occur in relatively shallow water where Proudman or Greenspan resonance can occur	shallower and wider continental shelves have a greater influence
water-level change and currents	periods of O(minutes) with rapid oscillatory change	periods of O(minutes) with rapid oscillatory change	gradual change in of O(hours)
region of impact	can influence an entire ocean basin and beyond	local to regional in nature	local to regional in nature

**Table 2.** List of meteotsunamis reported in the literature with maximum wave heights and, where applicable, the local name. The numbers refer to the locations shown in figure 2.

no.	location	max. height (m)	local name	reference
1	British Columbia and Washington State	0.17		[15]
2	San Diego, CA	0.05		[16]
3	Chicago, Great Lakes (USA)	3.0		[11]
4	Newfoundland, Canada	1		[17]
5	Boothbay, ME	4		[12,18]
6	NE Atlantic coast of USA	0.5		[4,19,20]
7	Daytona Beach, FL	3.0		[21]
8	eastern Gulf of Mexico	>3		[22,23]
9	Arraial do Cabo, Brazil	0.60		[24]
10	Buenos Aires coast, Argentina	0.62		[25]
11	Portugal/France	0.20		[26]
12	west coast, UK	0.40		[27,28]
13	Ireland	>1.0		[29]
14	Scotland, UK	0.20		[30]
15	southern North Sea	0.80	*zeebeer*	[31]
16	Baltic Sea	2.0	*seebär*	[6,32]
17	Finland, Baltic Sea	2.0		[33]
18	Balearic Islands	5.0	*rissaga, resaca*	[34,35,36]
19	Sicily, Italy	∼1.5	*marrobbio* (‘mad sea’)	[37]
20	Malta	∼1.0	*milghuba*	[38]
21	Croatia	6	*ščiga*	[36,39]
22	Greece	0.80		[40]
23	Black Sea	3.2		[41]
24	Odessa, Black Sea	2.0		[9]
25	Dwarskersbos, South Africa	2.9		[42]
26	west coast of India	0.40		[43]
27	Sri Lanka	0.20		[14]
28	Longkou Harbour, China	2.9		[44,45]
29	South Korea	1.6		[46]
30	Nagasaki Bay, Japan	4.8	*abiki, yota*	[47]
31	Kural Islands	0.37		[7]
32	Taiwan	0.5		[48]
33	West Australia	1.1		[14]
34	Burnie, Tasmania	0.6		[14]
35	New Zealand	1.0	*rissaga*	[49]

Meteotsunamis are considered as a multi-resonant phenomenon, where destructive events occur only when a coincidence of several crucial factors takes place at the same time [8]. These include the following. (i) Local weather systems which are able to efficiently transfer energy into the ocean. For example, this could include resonance conditions such as Proudman resonance ([13]; see §2a), where the moving speed of the atmospheric disturbance is equal to the local shallow water wave speed. (ii) The continental shelf and slope topography, which controls the amount of shoaling as the wave generated by the atmospheric disturbance in deep water moves onto the coast. (iii) The topography and geometry of the coastline (harbours, bays, river mouths, etc.), which could have a natural frequency similar to the incoming meteotsunami waves. All three of the conditions described above depend on the coastal topography and bathymetry as the speed of the shallow water waves is dependent on the water depths. Another major influence which will determine whether a destructive meteotsunami will be realized for a particular atmospheric disturbance is the timing in relation to the local water-level (tidal and mean sea-level) conditions. If the meteotsunami occurs at low water, depending on the tidal range, then the height of the meteotsunami wave may not cause widespread damage. By contrast, if the meteotsunami occurs at the local high tide level, together with a higher water level from a storm surge or high mean seasonal sea level, then a destructive tsunami event can be generated. For example, in June 2012, a meteotsunami contributed to the highest ever water level recorded at Fremantle, Western Australia, in over 115 years of record-keeping [14].

Meteotsunamis are a global phenomenon (figure 2 and table 2), with regions that have regular occurrences often naming the meteotsunamis in their local language (table 2). Recent collections of papers in special issues of journals [3,9] have made significant advances in the science of meteotsunamis. Major meteotsunamis (height>3 m) resulting in significant damage to coastal infrastructure are (figure 2 and table 2): 6 m at Vela Luka (Croatia), on 11 June 1978 [9]; 4.8 m at Nagasaki Bay, Japan [47], on 31 March 1979; 4 m at Boothbay Harbor on the coast of Maine, USA, on 28 October 2008 [18]; 3 m at some bays and inlets of Croatian islands on 25 June 2014 [9]; 3 m at Daytona Beach, FL, on 4 July 1992 [21]; and 3 m along the coastline of Chicago, IL, on 26 June 1954. However, even smaller meteotsunamis with lower wave heights have resulted in damage; for example, on 17 August 2014, a meteotsunami propagating along the mouth of the Swan River in Fremantle resulted in a ship breaking its moorings and impacting on a rail bridge (see §4).

Although meteotsunamis are generally a local phenomenon, there have been many events where a meteotsunami has been generated at different locations, sometimes separated by more than 1000 km, but originated from the same weather system. Examples include: teleconnections between the Balearic Islands and the Adriatic Sea on 29/30 June 2012 [50]; along the Atlantic coast of the USA on 13 June 2013, when the storm systems originated from the Great Lakes [19,20]; along the northwest European shelf from Portugal to the UK [26]; along the west coast of Australia on 13 January 2014, when a single thunderstorm created meteotsunamis along a 500 km stretch of coastline [14]; and when meteotsunamis were generated from the Balearic Islands to the Black Sea on 23–24 June 2014 [51].

In the analysis of sea-level time series, the usual practice is to use harmonic analysis to identify the tidal constituents (due to astronomical effects) and subtract the ‘tidal’ time series from the observed series to obtain the ‘residual’ or the ‘storm surge’ (e.g. [52]). Thus, in this type of analysis, all non-tidal components of the sea level were defined as storm surge. Recently, data analysts have separated the ‘residual’ component of the time-series signal from the storm surge component, defined as a signal having periods of more than 12 h subsequent to removal of the tidal component and the meteotsunami signal having periods of less than 6 h ([14]; see also §3). Reports of unusual coastal flood events, even during fair weather conditions, have been reported by locals but have gone unexplained. Traditional water-level records were sampled at 1 h intervals and were thus inadequate to examine/confirm the occurrence of meteotsunamis from measurements. Recent tsunami monitoring standard stations with sampling at 1 min intervals now allow for the identification of meteotsunami events globally. Therefore, although there is a wide distribution of reported meteotsunamis globally, their occurrence is perhaps more widely distributed than that depicted in figure 2. It should also be noted that there are many regions where major events have recurred due to particular local conditions such as local climate, topography and tidal range. As meteotsunamis have wave forms which are similar to seismic- and landslide-generated tsunamis, it is possible that some of the ‘tsunami-like’ events of ‘unknown origin’ which appear in some tsunami catalogues may be meteotsunamis [8]. It should be noted that there are other short period oscillatory water-level phenomena at the shoreline generated by wind-up and set-down [52,53].

Meteotsunamis are generated by meteorological events, particularly moving pressure disturbances. However, water levels respond to a range of meteorological forcing at different time scales: they range from storm surges (table 1), where the sea-level response is of the order of hours, to seasonal changes, of the order of months [54]. When we consider tsunami waves generated by seismic activity, every event is classified as a tsunami irrespective of the magnitude of the waves [8]. However, owing to different mechanisms and temporal scales of forcing through the atmosphere the definition of a meteotsunami is not very clear. Monserrat *et al.* [8] suggested a threshold criterion for an event to be classified as a meteotsunami as a wave amplitude which exceeded 4*σ, where *σ* is the standard deviation of the water-level residual time series (defined as that where the tidal and storm surge components have been removed).

Southwest Australia is a region that is impacted by both seismic and meteorological tsunamis (figure 1), and analysis of the sea-level records from several stations has indicated that meteotsunamis occur frequently in this region [14]. The region is impacted by a range of meteorological systems dominated by anti-cyclonic high-pressure systems with periodic tropical and mid-latitude depressions and local seasonal sea breezes [54]. Anti-cyclones move to the east and pass the coast every 3–10 days and, during winter, the region is subject to approximately 30 mid-latitude depressions and associated frontal systems [55]. Sea breezes dominate during the summer months with offshore (westward) winds in the morning and strong (up to 15 ms^−1^) shore parallel sea breezes commencing around noon and weakening during the night [56]. Regular thunderstorms are experienced in the afternoon during the summer months. The tides in the region are diurnal with a mean range of 0.5 m, with the range between lowest and highest water levels being 2.1 m [57]. Thus, a high percentage of the water-level variability is due to non-tidal components.

This paper reviews the occurrence, generation and enhancement of meteotsunamis globally and uses idealized numerical simulations and water-level data from southwest Australia to illustrate the different processes and an incident which occurred in August 2014 to highlight the hazard due to meteotsunamis. This paper is arranged as follows: §2 reviews the generation and enhancement of meteotsunamis. The numerical simulations and data analysis techniques are outlined in §3; results are presented in §4; and the discussion and conclusion are presented in §5.

## 2. Meteotsunamis: generation and enhancement

In the evaluation of seismic tsunami impact on coastlines, different stages of the tsunami wave development are considered, including generation source (e.g. magnitude of the earthquake slip), deep water propagation and coastal inundation. Similarly, for meteorological tsunamis, which are generated by a moving atmospheric pressure disturbance (jump), several processes are important to define the wave heights incident at the coastline. The magnitude of the pressure disturbance is of the order of 2–5 hPa, which corresponds to a water-level change of 0.02–0.05 m (inverse barometric effect [52]). The measured wave heights of meteotsunamis are much greater than those due to the inverse barometric effect alone (table 2). This indicates that there are additional processes which control the maximum wave height of meteotsunamis incident at the coast. These include (also applicable to seismic tsunamis) resonance conditions, continental shelf and slope topography and coastal geometry. There is also a major difference between the mode of wave propagation in seismic and meteotsunamis: seismic tsunami propagation is defined as a ‘free’ wave where the wave propagation is independent of the generating source (the earthquake). By contrast, meteotsunami waves are usually ‘forced’ waves linked to the moving atmospheric pressure disturbance, although waves could also travel as ‘free’ waves after the passage of the atmospheric pressure disturbance (e.g. in the wake of the storm [17]).

By definition, meteorological tsunamis are generated by a moving atmospheric pressure disturbance; usually, only a small change in pressure of less than 5 hPa over a 10 min period is sufficient to generate a meteotsunami. There are many different processes which result in a moving atmospheric pressure disturbance which have been documented to generate meteotsunamis: squalls [21,23,33], thunderstorms [14,58], frontal passages [14,30,51], tropical and extra-tropical storms [16,17,28,49,59] and atmospheric gravity waves [15,25,18]. It should be noted that the process which results in a moving pressure disturbance is often unique to a particular region.

In the following sections, different resonance effects which lead to the amplification of meteotsunami waves are reviewed briefly.

### (a) Proudman resonance

If we consider an atmospheric pressure disturbance *ΔP* moving with speed *U* in a constant water depth and solving the governing equations of linearized depth-averaged equations (neglecting friction, Coriolis and advection terms), the following expression may be derived (see [60]; ‘Proudman expression’ [13,51]):
2.1
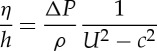
where *η* is the sea surface displacement and *ρ* is the seawater density. If *η*_s_=−(*ΔP*/*ρg*) is the stationary case ( *g* is the acceleration due to gravity), then
2.2
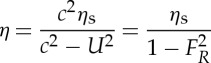
where *F*_*R*_=*U*/*c* is the Froude number. In the non-dimensional case,
2.3
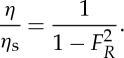
Thus for an atmospheric disturbance *ΔP*, moving with speed *U*, the surface displacement due to meteorological effects depends on the Froude number. From equation (2), for the case when the speed of the pressure disturbance is equal to the shallow water wave (*U*=*c*; *F*_*R*_=1), 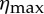 is unbounded due to lack of damping (e.g. friction) and a resonance condition exists. This is defined as the Proudman resonance [13]. However, in reality, due to friction and topographic effects, the ratio 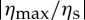, defined as the resonant factor *ε* [8], is limited and has been observed and predicted to reach a maximum value of 5 [47,61,35]. Typical speeds of atmospheric disturbances are 20–40 ms^−1^, which correspond to shallow water wave speeds in water depths of between 40 and 160 m.

When *F*_*R*_≪1, corresponding to deep water waves (*U*≪*c*), equation (2) yields [51]:
2.4
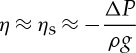
i.e. the surface water displacement is equivalent to the stationary case of the inverted barometric factor (1 *hPa*∼±1 cm). For *F*_*R*_≫1, (*U*≫*c*), a relatively higher speed of the atmospheric disturbance yields *η*≈0 [20]. This is due to the fact that the time interval for energy transfer from the propagating pressure disturbance is insufficient for an ocean response [60]. However, in the case where *F*_*R*_>1, defined as a supercritical storm, a wake similar to that behind a high-speed marine vessel is generated, resulting in meteotsunamis [62]. Another feature of the Proudman resonance is that when *F*_*R*_<1 atmospheric pressure and water displacement are out of phase with an elevation above mean level, while when *F*_*R*_<1 they are in phase when the wave is a depression.

For the case when *F*_*R*_≈1, where the atmospheric propagation is across a shelf with gradually varying water depth, Hibiya & Kajiura [47] derived the sea surface displacement as
2.5
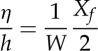
where *W* is the width of the air pressure disturbance and *X*_*f*_ is the distance travelled by the pressure disturbance across the shelf (‘fetch’). Therefore, the fetch or duration of the disturbance as well as the direction of propagation in relation to the orientation of the coastline are also important factors in generating meteotsunamis.

Proudman resonance has been suggested as the main cause of meteotsunamis occurring worldwide; for example, in Ciutadella Harbour, Balearic Islands [8,63,64], in Nagasaki Bay, Japan [47], along the Croatian coast in the Adriatic Sea [65], in southern UK [28,58], off the west coast of Korea [46], along the southwest Australian coast [14] and along the Gulf of Mexico [23].

### (b) Greenspan resonance

The continental shelf is able to contain the incident energy from offshore and/or from the atmosphere in an efficient manner to allow many ocean processes to be significantly amplified [66]. These include the amplification of tides through the quarter wave resonance [52] and continental shelf waves generated through tropical cyclones which are able to transport energy over large distances [67]. Edge waves are generated through reflection and refraction of energy within the continental shelf [68] and propagate along the shoreline in a packet form of time-varying finite extent. They are confined to a particular distance from the shore with the maximum amplitude at the shoreline. Observations of meteotsunamis at Scripps pier (California) in January 1954, described by Munk *et al.* [16], were attributed to the generation of edge waves along the coast due to storm systems. The analytical theory proposed by Munk *et al.* [16] for the steady state where the atmospheric pressure disturbance was present and was in motion for an infinite time interval was extended by Greenspan [69] to include the transient case. Using the linearized shallow water equations with a moving atmospheric pressure forcing Greenspan [69] obtained the surface elevation across the continental shelf as consisting of an infinite number of modes. On a plane beach with slope *β*, the edge wave dispersion relation is [70]
2.6
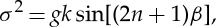
where *σ* is the edge wave frequency, *k* is the wavenumber and *n* is the mode number. The wave celerity is given by
2.7
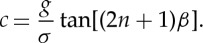
Greenspan resonance is defined as the condition when the speed of the atmospheric pressure disturbance in the alongshore direction is close to one of the modes of the edge wave propagation speed given by equation (7). In this case, the resultant edge wave heights are increased due to the resonance condition. The strength of resonant amplification is dependent on the propagation speed, amplitude of the pressure disturbance as well as the sea bed slope close to the shoreline. Edge waves, generated by a moving atmospheric pressure disturbance, parallel to the shoreline, have their crest lines perpendicular to the shoreline [71]. Numerical simulations using a three-dimensional numerical model have found that the maximum resonant amplification did not occur at the ‘critical’ resonant condition (defined as the speed of the pressure disturbance equal to the fundamental edge wave speed). Owing to the generation of harmonic edge wave modes, resonant amplification is maximized at supercritical conditions in which multiple edge wave modes superimpose to achieve a water-level fluctuation that exceeds that of the ‘critical’ resonant conditions [72].

Vilibić & Šepić [36] and Bechle & Wu [72], using numerical simulations, demonstrated that Greenspan resonance was responsible for the generation of meteotsunamis in the middle section of the Adriatic Coast and in the Great Lakes. However, many studies have concluded that both Proudman resonance and Greenspan resonance were responsible for the amplification of the meteotsunamis on the continental shelf (e.g. [23,72]).

### (c) Topographic resonance

We define topographic resonance as that resulting from natural oscillations of the continental shelf regions, bays and harbours. A free oscillation in an enclosed or semi-enclosed body of water, similar to the oscillation of a pendulum where the oscillation continues after the initial force has stopped, is defined as a seiche [73]. Several factors cause the initial displacement of water from a level surface, and the restoring force is gravity, which tends to maintain a level surface. Once formed, the oscillations are characteristic only of the system's geometry (length and depth) and may persist for many cycles before decaying under the influence of friction. The natural period of a continental shelf is given by Merian's formula for an open system [52]:
2.8
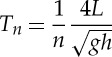
where *L* is the width of the continental shelf, *h* is the mean water depth, *g* is acceleration due to gravity and *n* is the mode number (i.e. 1 for the fundamental mode).

When the natural oscillating period of a continental shelf is equal to the periods contained in a meteotsunami, shelf resonance similar to that experienced with seismic tsunamis occurs [2,74,75].

In addition to the resonance phenomena described above, wave amplification and resonance oscillations are controlled by local topographic conditions on a continental shelf, bay or harbour including factors such as length/width, water depth, bed slope and topographic funnelling [8,36,61].

## 3. Methodology

### (a) Numerical simulations

**Figure 3.**
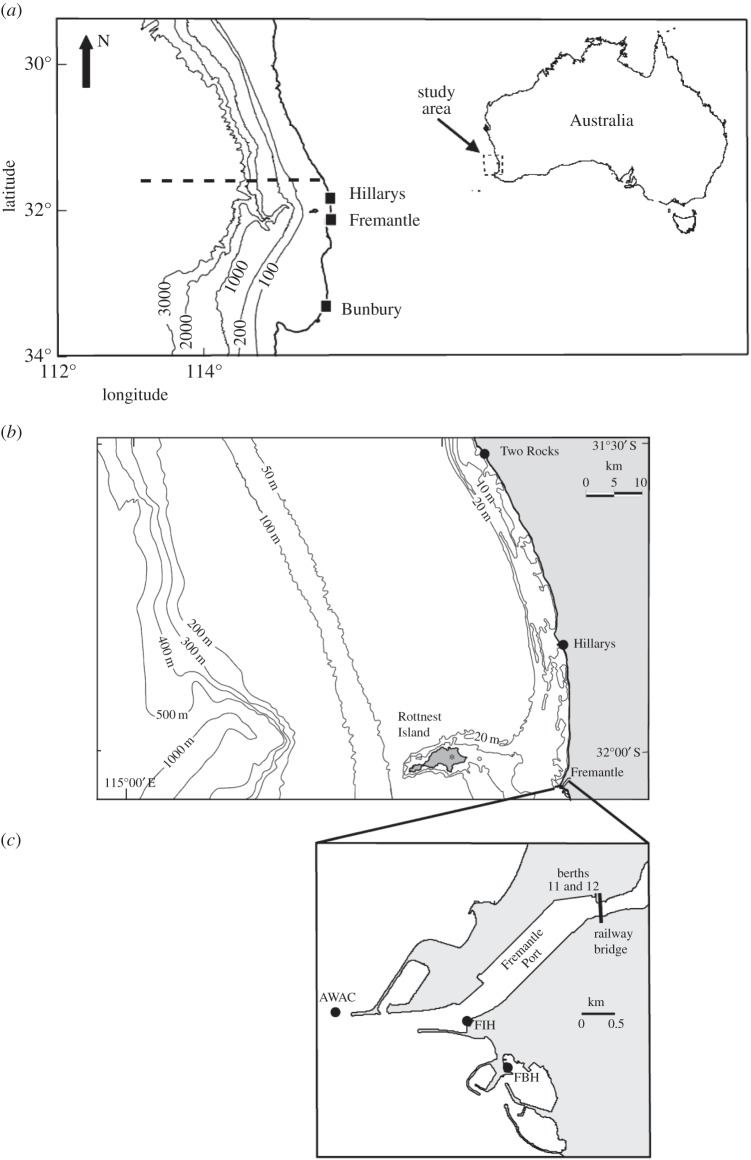
Maps showing the study region in southwest Australia with bathymetry in metres and locations of tide gauges (dots) and the meteorological station location at Rottnest Island (*b*). (*a*) Shows the numerical model extent. (*c*) Location of measurement stations in the vicinity of Fremantle Port. AWAC shows the location of current measurements. AWAC, acoustic wave and current; FBH, Freemantle Boat Harbour; FIH, Freemantle Inner Harbour.

Numerical simulations were undertaken using a two-dimensional, barotropic, nonlinear unstructured grid hydrodynamic model MIKE 21, developed by the Danish Hydraulic Institute. The model was forced by the specification of an idealized moving pressure disturbance. The model grids included an idealized long channel and offshore and continental shelf regions of southwest Australia (figure 3). Numerical simulations were undertaken using a depth-averaged, two-dimensional nonlinear finite-element hydrodynamic model, MIKE 21, developed by the Danish Hydraulic Institute (DHI). The following model configurations were used for the results presented in §4.

(1) To examine Proudman resonance conditions, an idealized narrow channel length of 420 km and width of 1.2 km was used. The grid resolution was 200 m. The water depth of the channel was constant within the domain but was set to vary for different model runs (between 15 and 95 m) to obtain a range of Proudman resonance conditions. The model was forced only by a pressure jump travelling at a constant speed of 20 ms^−1^ with an amplitude of 4 hPa varying over a distance of 50 km.
(2) To demonstrate the effects of shoaling across the continental slope, as the meteotsunami travels from offshore onto the continental shelf, a model set-up using the actual bathymetry of the southwest Australian coast was used. Grid resolution was chosen based on depth and coastal topography: the coastal region consisted of a finer grid with minimum resolution of approximately 200 m and relatively coarser grids (max. around 4000 m) in deeper waters. The model forcing was similar to the idealized channel case above, with forcing by a pressure jump with amplitude 4 hPa varying over a distance of 50 km. The direction of propagation and the speed of the pressure jump were changed to examine the sensitivity of the model for these parameters.

### (b) Data analysis

**Figure 4.**
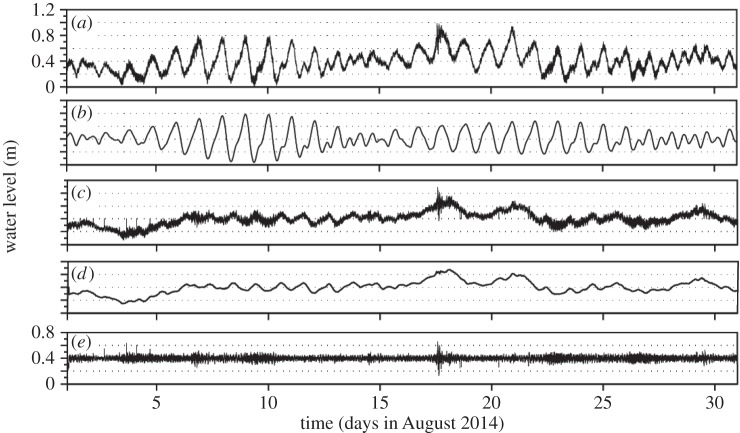
Time series of the Fremantle sea-level record for August 2014 showing the filtering procedure used to isolate the meteotsunamis with periods of less than 6 h: (*a*) the observed water-level time series; (*b*) the tidal component time series from the harmonic analysis; (*c*) the residual time series: observed record(*a*) – tidal component(*b*); (*d*) low-pass-filtered time series; and (*e*) time series with periods of less than 6 h to identify tsunami waves (both seismic and meteo): series(*c*) – series(*d*).

The annual time-series record for Hillarys Boat Harbour (figure 3), with a sampling interval of 1 min, collected in 2014 was analysed for this study. In addition, water-level data from Two Rocks, Fremantle Inner Harbour, Fremantle Boat Harbour and current meter data from inside and outside Fremantle Harbour for August 2014 were analysed. The corresponding meteorological data were also obtained from the Bureau of Meteorology station located at Rottnest Island (figure 3). The water-level time-series records were subjected to several filtering methods to isolate the meteotsunami signal (see figure 4, which shows the results obtained from Fremantle in January 2013):

(a) The observed water-level record (figure 4*a*) was subjected to harmonic analysis using the T-Tide Matlab toolbox [76] to remove the tidal components (figure 4*b*) from the sea-level records, resulting in the residual time series shown in figure 4*c*.
(b) The residual time series (figure 4*c*) was subjected to a low-pass filter to remove the periods less than 36 h, resulting in a time series that included the storm surge and weather band frequencies (figure 4*d*).
(c) The storm surge and weather band frequencies (figure 4*d*) were subtracted from the residual time series (figure 4*c*) to provide a time series that contained periods approximately less than 6 h, which included seiches and tsunami waves (both seismic and meteo).

The water-level time series were subjected to Fourier analysis to identify the dominant frequencies in the records and their variation with time through the construction of time–frequency plots. Here, time series of 4096 points at 1 min sampling intervals were used to estimate power spectra using the Welch method [77] using the fast Fourier transform method. Subsequent spectra were calculated using a 75% overlap.

## 4. Results

### (a) Proudman resonance

**Figure 5.**
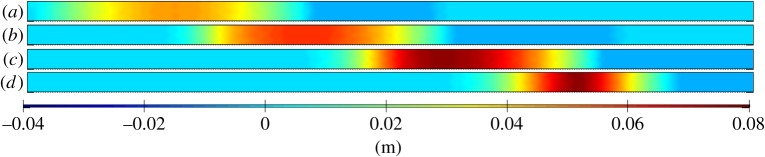
Snapshots of distribution of the water levels along a narrow, long channel. The channel is 400 km long and the snapshots are at 55 min intervals. Panels (*a*–*d*) represent water levels at 50 min intervals.

**Figure 6.**
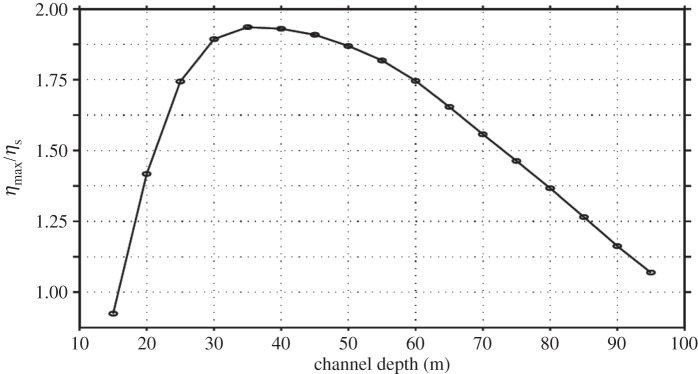
Variation in the resonance factor (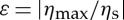) for different water depths along a narrow long channel.

Many of the previous studies (§2a) have indicated that Proudman resonance is one of the main mechanisms for the generation of meteotsunamis. An idealized one-dimensional example of Proudman resonance and its sensitivity to different combinations of the speed of moving pressure disturbance (*U*) and the shallow water wave speed (*c*) were investigated using a depth-averaged numerical model of a long narrow canal with a flat bottom (§3a, figure 5). The time series of the distribution of wave heights along the channel for the conditions close to when Proudman resonance is expected to occur indicated a progressive increase in the wave height as the pressure disturbance moved along the channel with time (figure 5). Changing the water depth (which in turn is related to the shallow water wave speed, *c*) indicated that the maximum response factor occurred when the water depth was approximately 40 m, i.e. when *U*=*c* (figure 6). In the propagation of the pressure disturbance along the canal, initially the maximum wave height was similar to the stationary case (inverted barometric factor) of approximately 0.04 m (*ε*=1), which increased to approximately 0.08 m (*ε*=2) approximately 3 h later towards the end of the channel (figure 5). Thus, Proudman resonance in this situation resulted in the doubling of the wave heights along the channel. Previous studies have indicated that *ε*<5 due to frictional and topographic effects [47,61,35], and these simulations agree with those findings. However, Whitmore & White [12] reported values of the resonance factor *ε*∼100 in a similar experiment, but with a shorter wavelength of the pressure jump (12 km compared with 50 km used here). This demonstrated that the resonance factor depends on the wavelength of the pressure disturbance. Vilibić [61] highlighted the importance of the wavelength of the pressure disturbance, particularly in narrow shelves, as shorter wavelength disturbances were more efficient in transferring the energy to the sea.

### (b) Wave shoaling

**Figure 7.**
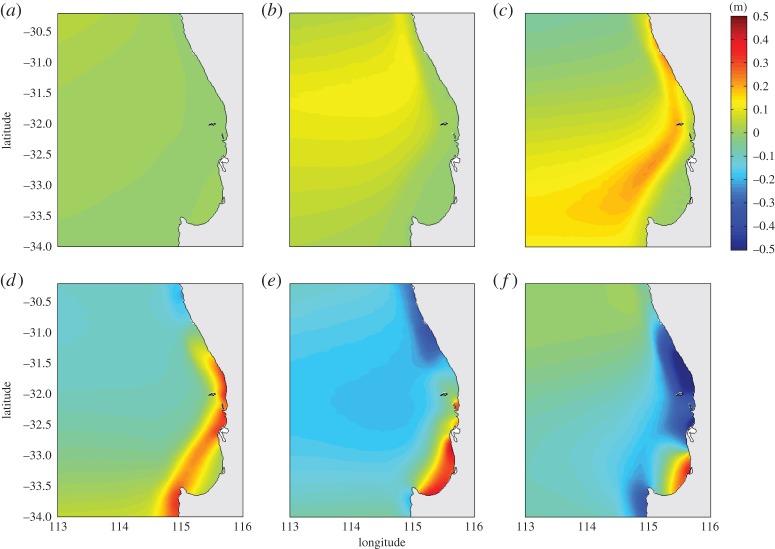
Time series of the predicted water levels generated by a pressure jump moving from north to south with orientation west to east. Panels (*a*–*f*) represent snapshots at hourly intervals.

In a region where the depth changes are uniform, wave shoaling based on linear wave theory is usually deemed to be governed by Green's law (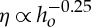, where *h*_*o*_ is the deep water depth; see also [78]). To examine the wave shoaling behaviour for meteotsunamis in southwestern Australia, a depth-averaged unstructured grid model with actual bathymetry was applied. The forcing was limited to a 4 hPa pressure jump moving with different speeds and directions. The variation of the water level, with time, for the case where the pressure jump is travelling from north to south highlighted the role of topography and wave refraction in this particular region (figure 7). Initially, the water-level changes (0.10 m; *ε*=2.5) are limited to deeper water only (figure 7*b*). As the pressure jump progresses southward, the waves to the north are aligned parallel to the shore due to refraction, while to the south, in deeper water, the increase in water level is oriented east–west, aligned with the pressure jump forcing. The maximum water levels are now 0.20 m (*ε*=5), with the highest values along the continental shelf break (figure 7*c*). When the pressure forcing has ceased (the pressure jump moved out of the model domain), the meteotsunami is present also on the shelf, in the central region of the domain (between latitudes 31.5° S and 32.5° S), while between latitudes 32.5° S and 33.5° S the meteotsunami is along the shelf break (figure 7*d*). The maximum water levels are now more than 0.4 m (*ε*>10). Subsequent time steps indicate the propagation of the meteotsunami onshore to Bunbury (figure 7*f*).

**Figure 8.**
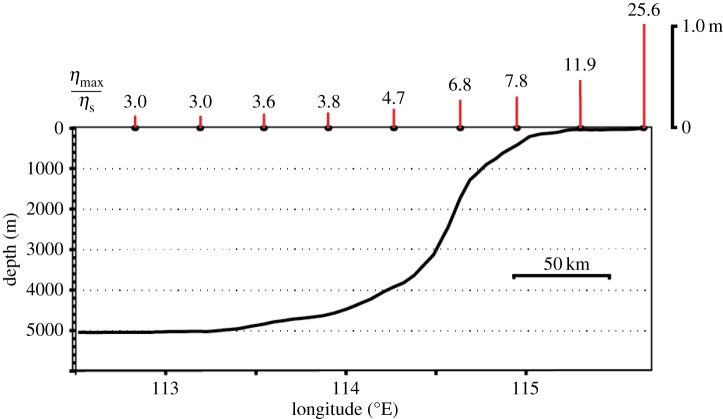
Variation in the resonance factor (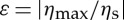) as the simulated meteotsunami wave propagates from deep water to nearshore. The location of the transect is shown in figure 3*a*.

The variation in the maximum wave height as the meteotsunami propagates from deep water across the continental slope and onto the continental shelf highlights the importance of the wave shoaling (figure 8). In deep water, the maximum wave heights were 0.12 m (*ε*=3) and increased slightly towards the shore with the shoaling process being most effective from approximately 2000 m water depth. Here, the wave heights increased more than threefold from 0.27 to 1.03 m (*ε*=6.8–25.6), a significant increase in the wave height from deep to shallow water (figure 8). These results are analogous to those reported by Hibiya & Kajiura [47] to explain the meteotsunamis (‘abiki’) in Nagasaki Bay, Japan. Here, the initial waves were generated by a moving pressure jump of 3 hPa across the east China shelf and were amplified (*ε*=4) through Proudman resonance over the initial 300 km, in water depths of 50–150 m. The amplification continued across the shelf slope, due to shoaling, for a resonance factor *ε*>40 at the harbour entrance and was estimated to be *ε*∼190 in the regions that were damaged [8,47].

**Figure 9.**
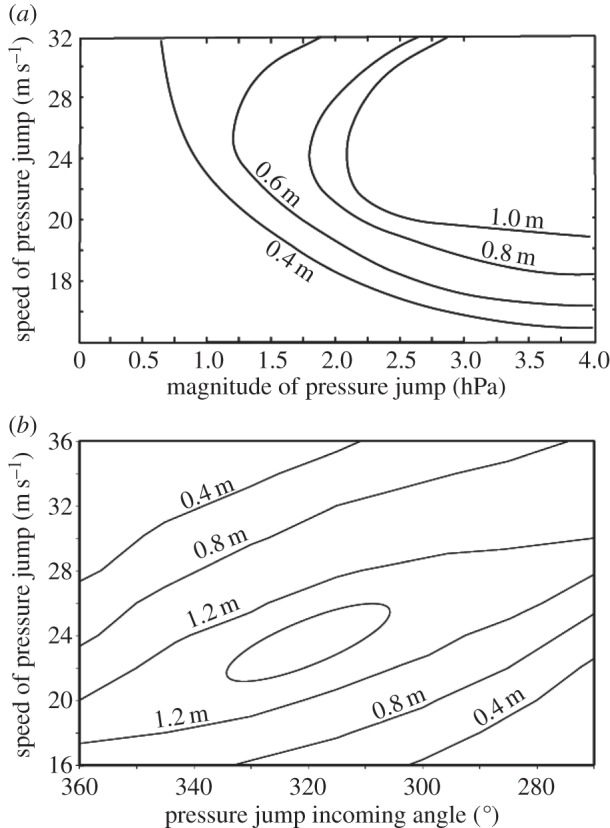
Predicted meteotsunami wave heights at Bunbury (figure 3) using idealized forcing: (*a*) Maximum predicted wave heights due to a moving pressure jump, moving from north to south with different magnitude and speed and (*b*) Maximum predicted wave heights due to a 3 hPa moving pressure jump with different magnitudes and directions.

Several model runs were undertaken (with actual bathymetry) with the different magnitudes, propagation speeds and directions and the predicted maximum wave heights were extracted for Bunbury (figure 1). The model results indicated that all three parameters (magnitude, speed and direction) influence the maximum wave height predicted at Bunbury (figure 9). For a particular propagation direction, larger maximum wave heights were generated by a higher pressure jump with travelling speeds between 20 and 32 ms^−1^ (figure 9*a*). Propagation speeds of less than 20 ms^−1^ resulted in lower wave heights for the same magnitude of the pressure jump. The maximum wave heights were generated when the air pressure disturbance was travelling from direction 310°–340° (from NNW) at speeds between 20 and 24 ms^−1^ (figure 9*b*); however, higher and lower propagation speeds produced smaller wave heights. Previous studies [79,80] also highlighted the influence of the direction of the pressure disturbance propagation on the maximum wave heights. This was attributed to the distance travelled over the shelf, which influenced wave amplification towards the coast, through Proudman resonance and ‘fetch’ (see equation (5)).

### (c) Meteotsunamis in southwest Australia, 2014

**Figure 10.**
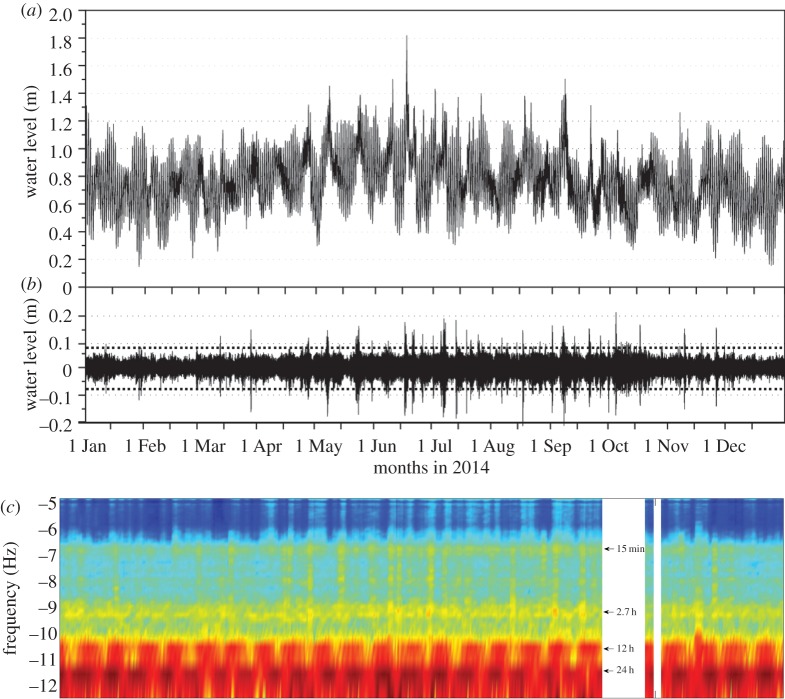
Time series of the Hillarys (figure 3) sea-level record for 2014 with sampling at 1 min intervals; (*b*) filtered water level (less than 6 h period) showing the meteotsunamis. The dashed line shows the 4*σ value. (*c*) Time–frequency diagram obtained through Fourier analysis. The time axis is common to all three panels.

**Figure 11.**
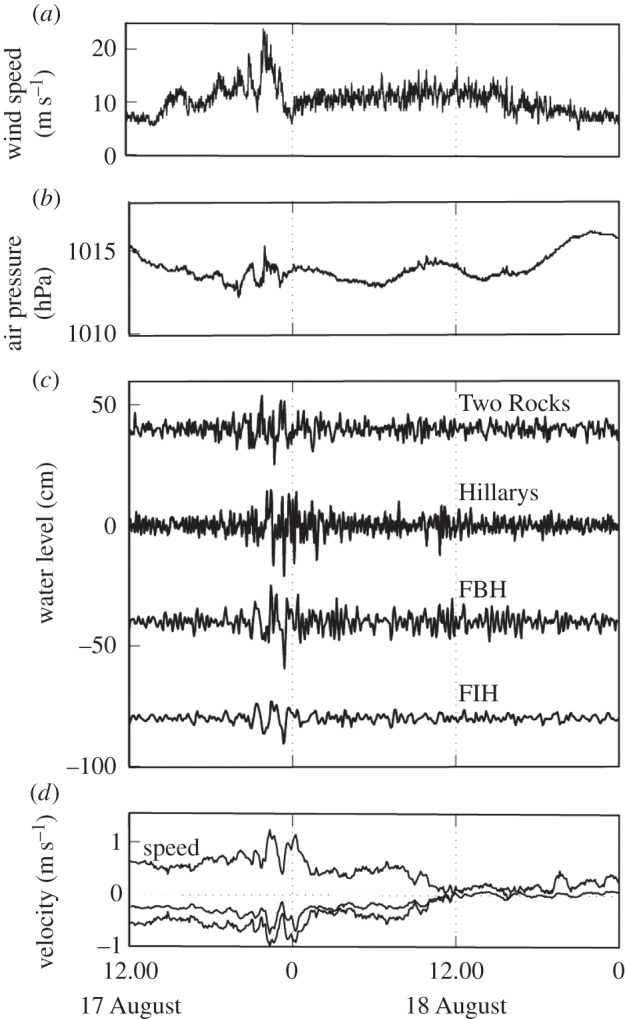
Time series of meteorological and oceanographic data associated with the meteotsunami in Fremantle Port on 17 August 2014. (*a*,*b*) Wind speed and atmospheric pressure at Rottnest Island; (*c*) filtered water levels at Two Rocks, Hillarys, Fremantle Boat Harbour (FBH) and Fremantle Inner Harbour (FIH); and (*d*) measured currents at AWAC station. Locations are given in figure 3.

The annual sea-level record for Hillarys in 2014 exhibits typical sea-level characteristics as reported in the literature (figure 10*a*): there is a fortnightly cycle of tropic and equatorial tides with storm surge and continental shelf wave signals superimposed. The mean sea level is a maximum during the austral winter due to oceanic processes [54]. The maximum water level was 1.82 m during the passage of the cold front in mid-June (figure 10*a*). The sea-level record from Hillarys, with a sampling interval of 1 min, was subject to the sequence of filtering as described in §3b to extract the time series with periods less than 6 h (figure 10*b*) and time–frequency analysis (figure 10*c*). Using the threshold criterion suggested by Monserrat *et al.* [8] to classify a meteotsunami as a wave amplitude which exceeds 4*σ, in 2014, there were more than 30 events recorded in the time series with the majority in terms of magnitude and number occurring during the winter months. The maximum recorded wave height was 0.4 m on 5 October. The event on 17 August (see §4d) had a maximum wave height of 0.35 m (see also figure 11). The occurrence of a higher number of events during the winter months is mainly due to the passage of mid-latitude depressions and associated frontal systems. The time–frequency analysis reveals the different frequencies in the record and their changes with time. The tidal frequencies and their fortnightly modulation are clearly present at the 24 h (diurnal) and 12 h (semi-diurnal) frequencies (figure 10*c*). Other frequencies at 2.7 h and 15 min are due to local seiches. The spectral energy at the 2.7 h oscillation is present almost all the time and has been attributed to the continental shelf seiche (see §2c and equation (8)) with the continental shelf width being approximately 50 km. The 15 min oscillation is due to the presence of a limestone reef system offshore of Hillarys Marina, where the tide gauge is located. Here, the mean water depth of approximately 16 m results in a period of approximately 15 min using equation (8). A similar period of oscillation (13 min) was reported by Thotagamuwage & Pattiaratchi [81] at Two Rocks (figure 1). A feature of these two oscillations (and perhaps another minor one at approx. 1 h) is that there is energy at both of these frequencies almost continuously throughout the year. These represent the background oscillations in the filtered time-series record which fall into the category of less than 4*σ and thus are not classified as a meteotsunami (figure 10*b*). However, there are periods when the energy is enhanced, coinciding with the meteotsunamis: it appears that, during the passage of a frontal system, the whole spectrum is energized, as shown in the higher energy bands across all frequencies, which correspond to the meteotsunami events (cf. figure 10*b*,*c*). This increase in energy across all the frequencies and that enhances the existing frequencies was reported for meteotsunamis at other locations along western Australia [14] and for seismic tsunamis along western Australia and Sri Lanka [74].

### (d) Meteotsunami on 17 August 2014

At 2203 h on Sunday 17 August 2014, car carrier *Grand Pioneer* and the container ship *AAL Fremantle* were moored in Fremantle Port at berths 11 and 12 (figure 3*c*), respectively. A bollard that was holding all three of the *AAL Fremantle*'s stern lines and two from the car carrier *Grand Pioneer* ripped off the wharf, causing both ships to swing away from berth. *AAL Fremantle*, freed from its stern lines, swung around and collided with the railway bridge (figure 3*c*), which was badly damaged and closed for two weeks, severely disrupting one of the major commuter railway lines in Perth, Australia. Initially, the incident was attributed to strong winds associated with a passage of a front, but further analysis revealed that the ship's moorings were broken by strong currents within the harbour which could be attributed to a meteotsunami. At the time of the incident, widespread thunderstorms were experienced in the region. Data from a local meteorological station at Rottnest Island and coastal water-level data from four locations were examined to determine the cause of the strong currents inside the harbour. Time series of atmospheric pressure indicated a gradual decrease, with two pressure jumps evident in the record (figure 11*b*). The first pressure jump of amplitude 2.1 hPa occurred over a period of 83 min (between 20.20 and 21.43) and was associated with a pulse of wind to a maximum speed of 17 ms^−1^; the second pressure jump was more severe, with a 2.4 hPa change over 14 min (between 21.43 and 21.57) with wind speeds up to 23 ms^−1^ and gusts up to 30 ms^−1^. Tide gauge records all indicted the presence of higher water-level fluctuations coinciding with the passage of the pressure jump. The higher water-level fluctuations were first observed at the northern-most station, Two Rocks, which is located 70 km away from Fremantle (figure 3*b*), and progressed southwards, in the direction of the pressure jump. The rainfall radar also indicated progression of the rain bands from north to south. The maximum wave heights at Hillarys and Fremantle (at both Inner and Boat Harbours, figure 3*c*) were observed 8 and 20 min later than those observed at Two Rocks, respectively (figure 11*c*). As the wave progressed in the harbour, very strong currents greater than 1.0 ms^−1^ (depth-mean) were measured to the north of the entrance breakwater (figure 3*c*), travelling in a southwesterly direction prior to entering the harbour. As the wave progressed inside the harbour, the constriction at the location of the bridge enhanced the currents at berths 11 and 12 (figure 3*c*) and resulted in the moorings being broken. The location of a shallow shoal, the Wangara shoal, immediately downstream of the railway bridge was thought to prevent ships impacting on the bridge. However, the water levels were higher due to the meteotsunami and thus the *AAL Fremantle* was able to pass over the shoal, although there was insufficient water after the impact with the bridge and a different route was used to relocate the ship at the berth.

Examining the sea-level time series for 2014, the event on August 2014 was not the largest event recorded during the year (figure 10*b*). It is also interesting that another event on 10 September, although not very large, resulted in the breaking of mooring lines within the port without any further damage.

## 5. Discussion and conclusion

Meteotsunamis are generated by meteorological events, particularly moving pressure disturbances due to squalls, thunderstorms, frontal passages and atmospheric gravity waves. Relatively small initial sea-level perturbations, of the order of a few centimetres, can increase significantly through multi-resonant phenomena to create destructive events through the superposition of different factors. Results from numerical modelling and field measurements from southwest Australia, presented in this study and by others, have demonstrated that meteotsunamis are initiated mainly through Proudman or Greenspan resonance. However, the main influence that leads to amplification of the initial disturbance is due to wave shoaling and topographic resonance.

The discovery and documentation of meteotsunamis in recent years have benefitted from developments in measurement and analysis techniques. Historical water-level records (usually analogue readings) were sampled at 1 h intervals to obtain the tidal, storm surge and long-term characteristics. This sampling interval was not optimum for identification of meteotsunami waves. If there was a report of an ‘unusual’ water-level event, it could not be analysed in detail even if there was a tide gauge in close proximity due to the sampling resolution. As the archived data are also at 1 h sampling intervals, it is not possible to re-visit historic events. Since the 2004 Indian Ocean mega-tsunami, the establishment of the tsunami warning systems has significantly increased the number of tide gauges globally (http://www.ioc-sealevelmonitoring.org/) as well as standardizing the sampling interval to 1 min, which allows for detailed analysis of the sea-level time-series records. The addition of alternative techniques such as high-frequency radar [82] to traditional tide gauges is also a new development. Quality control procedures for sea-level measurements, primarily designed to measure tides and long-term changes, flagged that any value greater than 3*σ (where *σ* is calculated from the residual time series) is to be defined as spurious and removed from the record. This criterion is lower than the 4*σ proposed by Monserrat *et al.* [8] to define a meteotsunami, and therefore it is possible that some meteotsunami events may not be present in the historical digitized datasets, although archived analogue records may contain records of meteotsunami events.

Since the 2004 Indian Ocean tsunami, there have been vast developments in the prediction of wave heights and inundation potential arising from seismic tsunamis and include modelling platforms such as ComMIT [83]. Here, the wave heights resulting from a seismic tsunami of a particular magnitude are predicted using information on the earthquake characteristics which generate the tsunami. By contrast, prediction of meteotsunamis is in the early stages and is dependent on the availability of high spatial and temporal resolution atmospheric models to be able to predict the exact location as well as the speed, amplitude and propagation direction of the moving pressure disturbance. To date, there have been many approaches but with mixed results. The only ‘operational’ system appears to be that of the Balearic Meteorological Service, which raises an alert if the synoptic atmospheric conditions are similar to those observed during previous meteotsunami events and through monitoring of sea-level stations [84]. The ability to predict the exact weather system (e.g. thunderstorm, squall) at fine temporal and spatial resolution is inhibited by the availability of meteorological data, particularly air pressure, in sufficient resolution to be assimilated into the model. Also standard meteorological forecast output at 3-hourly intervals is inadequate to capture the pressure change of approximately 0.3 hPa min^−1^ required to generate a meteotsunami [85]. Thus, many of the proposed prediction systems have used meso-scale weather predictions to identify conditions when a meteotsunami may be generated [50,86,87]. An application of the Weather Research Forecast atmospheric model to the Balearic Sea region was able to reproduce the development of a convective nucleus and speed of the atmospheric pressure disturbance [85]. Recently, the TMEWS (Towards a MEteotsunami Warning System) project examined options for the development of a meteotsunami warning system along the US coastline [9].

In conclusion, are meteotsunamis an under-rated hazard? The documented evidence to date, presented here, has identified specific locations where destructive meteotsunamis occur as a combination of multi-resonance conditions. However, compared with seismic mega-tsunamis, loss of life and damage to infrastructure has been significantly lower. Seismic tsunamis are relatively infrequent highly energetic events able to create destruction across ocean basins. By contrast, atmospheric disturbances of various types (passing fronts, squalls and trains of atmospheric waves) are common and are able to generate meteotsunamis more frequently but that are much less energetic than seismic tsunamis. High-energy events occur only for very specific combinations of resonant effects. The rareness of such combinations is perhaps the main reason why destructive meteotsunamis are exceptional and observed only at a limited number of sites globally.

## Data accessibility

The field observations presented in this study were obtained from a variety of sources: (i) meteorological data were obtained from the Australian Government Bureau of Meteorology through payment of a fee; (ii) sea-level and current metre data from Fremantle Port were supplied by Fremantle Ports and are archived by Fremantle Ports; (iii) sea-level data from Fremantle Boat Harbour and Two Rocks were obtained by the Department of Transport (DoT), Government of Western Australia, through a research licence and are archived by the DoT; and (iv) sea-level data from Hillarys Harbour were obtained through the following website: http://www.ioc-sealevelmonitoring.org/.
